# Help-Seeking from a National Youth Helpline in Australia: An Analysis of Kids Helpline Contacts

**DOI:** 10.3390/ijerph18116024

**Published:** 2021-06-03

**Authors:** David Watling, Samantha Batchelor, Brian Collyer, Sharna Mathieu, Victoria Ross, Susan H. Spence, Kairi Kõlves

**Affiliations:** 1 W.H.O Collaborating Centre for Research and Training in Suicide Prevention, Australian Institute for Suicide Research and Prevention, School of Applied Psychology, Griffith University, Brisbane 4122, Australia; david.watling@griffithuni.edu.au (D.W.); s.mathieu@griffith.edu.au (S.M.); victoria.ross@griffith.edu.au (V.R.); s.spence@griffith.edu.au (S.H.S.); 2 Advocacy and Research Division, Yourtown, Brisbane 4064, Australia; sbatchelor@yourtown.com.au (S.B.); bcollyer@yourtown.com.au (B.C.)

**Keywords:** youth helpline, crisis hotline, telephone counselling, online counselling, help-seeking, young people, adolescence

## Abstract

Counselling helplines or hotlines are key support services for young people with mental health concerns or in suicide and self-harm crises. We aimed to describe young peoples’ use of a national youth helpline (Kids Helpline, Australia, KHL) to understand how usage changed over time. A descriptive analysis was conducted on 1,415,228 answered contacts between 2012–2018. We described the trend of service usage over the observed period, the types of youth who used the service, and the problems young people contacted the service about. Phone (APC = −9.1, KHL: −10.4 to −7.8, *p* < 0.001) and email (APC = −13.7, 95%CI: −17.1 to −10.2, *p* < 0.001) contacts decreased over time whereas webchat contacts increased (APC = 16.7, 95%CI: 11.7 to 22.0, *p* < 0.001). With this increase in webchat contacts, there was an associated increase in total webchat contact duration. Concerns raised in contacts to the service were primarily related to emotional wellbeing and mental health concerns (53.2% phone, 57.3% webchat, 58.2% email) followed by social relationship issues (20.4% phone, 20.3% webchat, 16.8% email) and family relationships (19.4% phone, 17.2% webchat, 21.8% email). The increased preference for online text-based information and counselling services can help inform development of services for young people and allocation of staff/service training and resources.

## 1. Introduction

Many mental health conditions first present at a young age (e.g., anxiety) [1]. A recent Australian study reported that nearly one in seven (13.9%) young people aged 4–17 years were assessed as having a mental health disorder in the previous 12 months [2]. Moreover, suicide mortality increases through the teenage years, with suicide remaining a leading cause of death in children and young adults [3,4,5]. Despite this, help-seeking and treatment utilization is often low [6,7]. According to a recent systematic review of young peoples’ perceived barriers to help-seeking for emotional well-being and mental health concerns, barriers were related to individual factors (e.g., mental health and services literacy), social factors (e.g., perceived stigma), relationship factors (e.g., fears surrounding confidentiality), and systemic/structural factors (e.g., access/time/transport and costs) [6].

Helplines can be a critical first point of contact in the prevention and care for young people with psychosocial concerns and those at risk of suicide. They provide confidential information and emotional support for people with various psychosocial concerns (e.g., emotional wellbeing, violence and abuse, social relationships [8]) and crisis intervention for individuals experiencing suicidal crisis [9]. By providing support through telephone, email, SMS, or online chat [10], helplines offer multiple advantages for users compared to face-to-face services, potentially addressing several barriers to help-seeking. For example, the confidentiality/anonymity of helplines may overcome perceived stigma, and helplines are also highly accessible and cost-effective. Furthermore, the introduction of online and text-based helpline modalities may also enhance help-seeking in young people [11,12].

Regarding the effectiveness of youth helplines around the world, a recent systematic review described the state of the literature and, despite noting a lack of empirical studies, suggested that youth helplines may effectively reduce immediate distress [13]. In addition, Mathieu et al. [13] concluded that relatively limited previous research has explored the reasons young people contact youth helplines, described up-to-date demographic features of those who use the services, or explored the use and effectiveness of different service contact media (e.g., phone vs. webchat). Identifying who, how, and why youth use helplines would help to tailor services to the needs and preferences of young people and contribute to improving their experience with and outcomes from engaging with helplines. 

The current study aimed to analyse changes in help-seeking behaviour through the Kids Helpline (KHL), a nationwide youth service in Australia from 2012–2018. We explored service utilisation, including the medium of contact, the reasons children, and young people (5–25 years) contacted the service and how use of contact media changed over time. 

Specifically, our research questions were:

(1) How many contacts did the service respond to through different contact media and by contact type (information and referral, counselling) and how has this changed over time?
(2) What are the differences by gender and age across media and by contact type?
(3) What is the duration of the different contact types and has it changed over time?
(4) What are the concerns of Australian children, do they differ across media, and did this change over time?

## 2. Materials and Methods

### 2.1. Kids Helpline (KHL)

Established in 1991, KHL provides young people aged 5–25 years with free 24/7 information and counselling accessible through telephone, webchat, and email. The KHL service model uses a stepped-intervention framework, providing a range of support options from ‘universal care’, in which young people are provided with information or short-term support for a simple problem, to ‘complex care’ for those with acute, chronic, and often multiple presenting issues such as complex mental ill health or risk of harm to self or others. Support is calibrated to respond to the needs of each individual and includes provision of information, referral to other services, psychoeducation, counselling, case planning, risk of harm assessment, and for those with the most complex needs, wrap-around care with other services. Counsellors are degree-qualified in psychology, social work, counselling, human services or similar, with at least one year of experience when recruited. Comprehensive in–house training provides additional understanding of specific issues and evidence–informed responses relevant to KHL. For the data collection period, all counsellors received one full day of training exclusively in the coding system and recording of data, this included approximately three hours on coding the ‘problem’ or concern raised during the KHL contact whereby counsellors discussed case examples in small groups before large group consensus.

### 2.2. Data Collection

The de-identified contact data analysed in this study were recorded by KHL counsellors between January 1, 2012 and December 31, 2018 as part of routine service delivery. The justification for the 2012 start date is that the coding system was revised at this time and there were differences in the data collected prior. More recent data were also not included in the current analyses, as the unprecedented impact of the global novel coronavirus disease (COVID-19) pandemic warranted a more granular analysis of weekly and monthly changes in demand (as opposed to annual), which have been reported elsewhere [14]. Furthermore, there were increases in web-chat service delivery from late 2018 onwards that may have unduly impacted the results. For each contact, counsellors record the client’s demographic details (e.g., age, identified gender, living arrangement) and other information about the contact (e.g., concerns raised by the young person) in an online platform. For reasons of confidentiality, counsellors do not specifically request demographic details for the purpose of data collection. Age is generally disclosed by the young person as part of a counselling conversation or inferred through other indicators (e.g., school grade). Gender (male, female, transgender, or gender diverse) is either inferred based on the interaction or asked at an appropriate time. Other details (e.g., living arrangement) are only recorded if the information arises in conversation. Consequently, age and gender are more likely to have been recorded for repeat than single callers. For the current study, young people have been separated into age groups, with ‘children’ aged 5–12 years, ‘teens’ aged 13–17 years, and ‘young adults’ aged 18–25 years.

Contact type is distinguished as either ‘counselling’ (i.e., contact focused on a specific concern), ‘information and referral’ (e.g., a request for information or contact details of a specific service), or ‘other ways of engaging’ (i.e., non-conversational, roleplaying a problem, and nonsensical, aggressive, or sexual ‘prank’ contacts). Counsellors record up to four concerns for each counselling contact using the KHL Concern Classification System [15], which is a list of 49 concerns (e.g., mental health concerns, bullying, suicidal thoughts, sexual abuse) grouped into 11 broader concern groups (e.g., family relationships, identity and self-concept, violence and abuse). Concerns reflect reasons for contact, that is, the issues the young person wished to speak about, not the counsellor’s perception of the young person’s most important problems.

### 2.3. Ethics

The study was approved by the Griffith University’s Human Research Ethics Committee (reference number: 2020/505).

### 2.4. Statistical Analysis

Dependent variables included the total number of contacts, contacts per medium (phone, email, webchat), concern type, and the number of contacts across gender (male, female, transgender or gender diverse) and age group (children, teens, young adults). The duration of webchat and phone contacts (seconds, minutes) was also analysed, but duration of email contacts was not, as email exchanges may occur over multiple days. Time trend analyses of dependent variables were analysed from 2012 to 2018.

Time trends were analysed using Joinpoint regression, which identifies time points where a statistically significant change in trend occurs. Joinpoint provides an estimate of the average annual percentage change (APC), with 95% confidence intervals (95% CI). Chi-square analyses were used to compare categorical data. IBM SPSS Statistics for Windows version 26 (IBM Corp, Armonk, NY, USA) and Joinpoint Regression Program version 4.8.0.1 (National Cancer Institute, Bethesda, MD, USA) were used to analyse the data.

## 3. Results

Between 1 January 2012 and 31 December 2018, 1,415,228 contacts to the KHL service were recorded. For further analyses some data were excluded. First, ‘Other’ contacts (35.9%, *n* = 507,648) were excluded as these did not inform the aims of the study. Second, contacts made by people outside the target age range (i.e., 5–25 years) were excluded (2.1%, *n* = 19,004), as were contacts where age could not be determined conclusively (20.6%, *n* = 182,424). Third, phone counselling contacts lasting less than 60 seconds were excluded (0.2%, *n* = 1462) following discussion with KHL staff, which indicated these do not represent meaningful counselling conversations. ‘Information and referral’ contacts of all lengths were included as the study team deemed these contacts were relevant, regardless of duration. 

**Figure 1:**
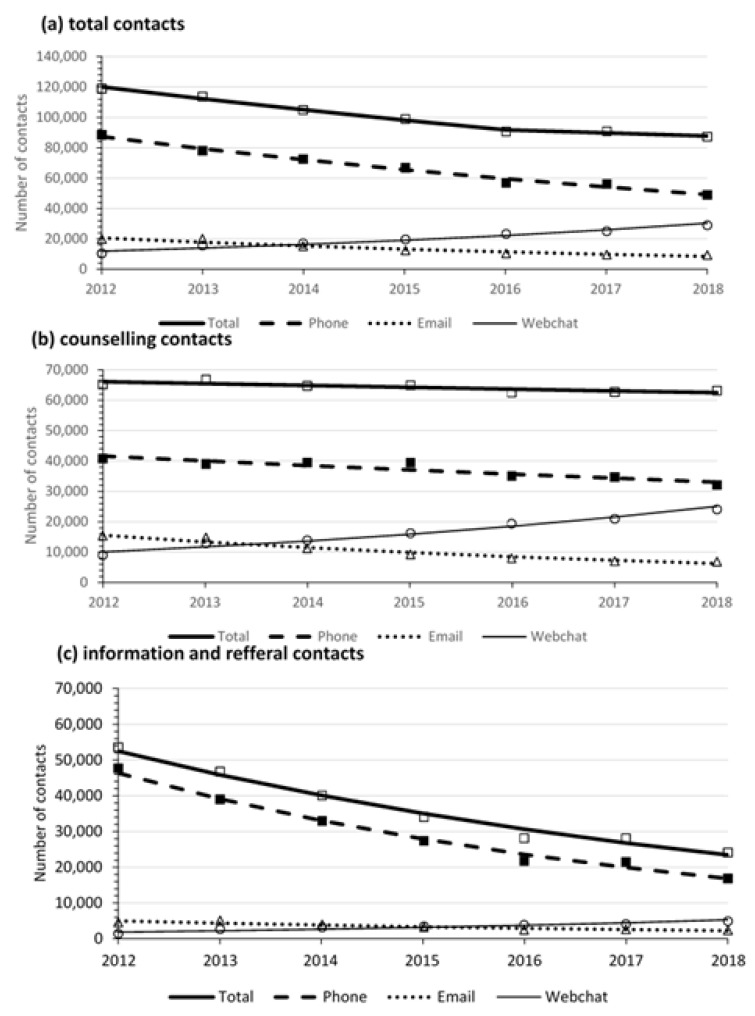
Number of contacts to KHL service in 2012–2018: (**a**) total, (**b**) counselling contacts, (**c**) information and referral contacts.

The following analysis includes 704,690 contacts. KHL allows young people to remain anonymous; nevertheless, many of those who contact multiple times identify themselves with a name or pseudonym. Based on these names, and matching of phone numbers, IP addresses and email addresses, approximately 35% (*n* = 24,745) of contacts were from a young person who made a single contact and approximately 65% (*n* = 457,445) were from a person who made two or more contacts. The total number of contacts decreased over the study period, with the APC showing a decline of 6.5% per year on average (95%CI: −8.3, −14.8, *p* = 0.005) between 2012 and 2016, followed by a non-significant decline from 2016–2018 (Figure 1). This primarily reflected a reduction in information seeking rather than counselling contact types.

### 3.1. Medium of Contact

Most contacts (66.4%, *n* = 467,626) were by phone, with 19.9% (*n* = 139,999) by webchat and 13.7% (*n* = 97,065) by email. Trends of contacts by medium and contact type from 2012–2018 are provided in Figure 1 and Joinpoint trend analyses in Table 1. In terms of contact medium, the number of phone (APC = −9.1%, 95%CI: −10.4, −7.8, *p* < 0.001) and email contacts decreased (APC = −13.7%, 95%CI: −17.1, −10.2, *p* < 0.001), whereas webchat contacts showed a rapid increase (APC = 16.7%, 95%CI: 11.7, 22.0, *p* < 0.001) over the study period.

### 3.2. Medium of Contact: Age, Gender, and Contact Types

Each contact’s age, reported gender, and contact type for phone, webchat, and email are presented in Table 2. Contacts by phone were primarily from young adults (53.4%), whereas contacts by webchat and email were primarily from teens (63.8% and 67.4%, respectively). Most phone contacts from young adults were for information and referral purposes, whereas phone contacts from teens and children were primarily for counselling. Across all media, most contacts were from those whose gender was recorded as female. Counselling contacts were the primary contact type across all media (63.8% of all contacts). However, the proportion of contacts that were counselling related (versus information and referral) was greater for webchat (83.3%) compared to the proportion of counselling contacts made by either phone (55.7%) or email (70%).

### 3.3. Information and Referral Contacts

A breakdown of information and referral contacts across media, age and gender are provided in Table 2. A total of 254,876 (36.2%) contacts were requesting information and/or referral. Information and referral contacts decreased over time (APC = −12.6, 95%CI: −14.7, −10.5, *p* = 0.001). Most were made by phone (81.3%, *n* = 207,113), followed by email (9.5%, *n* = 24,322), and webchat (9.2%, *n* = 23,441). Information and referral contacts made by phone (APC = −15.6, 95%CI: −17.6, −13.4, *p* <0.001) and email (APC = −12.7, 95%CI: −17.3, −7.8, *p* = 0.001) decreased between 2012 and 2018, whereas webchat contacts increased for this contact type (APC = 19.1, 95%CI: 8.4, 30.9, *p* = 0.005; see Figure 1 and Table 1). 

Information and referral contacts were more likely to be from young adults (56.4%, *n* = 143,677) than teens (34.6%, *n* = 88,226) or children (9.0%, *n* = 22,973) (Table 2). The age breakdown varied for each contact medium. Relatively more children made information and referral contacts by email, whereas relatively more teens made contact by webchat and email, and relatively more young adults made contact by phone. Most information and referral contacts were with girls (72.9%, *n* = 183,690) and fewer were with boys (25.5%, *n* = 64,238) or transgender or gender diverse young people (1.6%, *n* = 4044). The trend of gender breakdown was consistent across all media; however, relatively fewer contacts were made by boys through webchat and email compared girls and transgender or gender diverse young people

### 3.4. Duration of Information and Referral Contacts

The median duration of phone information and referral contacts across all years was just over two minutes (Table S1). The change in median durations between 2012 and 2018 was less than one minute (2012 = 1.92 minutes, 2018 = 2.72 minutes), which reflected a non-significant increase in duration (APC = 5.21, 95%CI: −0.1, 10.8, *p* = 0.052). As a measure of load on KHL services, the *total* duration of information and referral contact time per year by phone decreased significantly from 346,593 minutes in 2012 to 107,674 minutes in 2018 (APC = −18.48, 95%CI: −24.5, −12.0, *p* = 0.001). 

Regarding contact medium, the median duration of information and referral contacts made by webchat across all years was 8 minutes (Table S2), demonstrating a significant decline from 11.5 minutes in 2012 to 6 minutes in 2018 (Table S2; APC = −9.21, 95%CI: −11.7, −6.7, *p* < 0.001). However, regarding load on KHL services, there was a non-significant increase in total webchat information and referral contact time per year from 28,627 minutes in 2012 to 52,871 minutes in 2018 (APC = 5.99, 95%CI: −2.3, 15.0, *p* = 0.125). While non-significant, this near double in total load reflects the increase in number of webchat contacts made over time (Table S2).

### 3.5. Counselling Contacts 

A breakdown of counselling contacts across age, gender, and contact medium are provided in Table 2. There were 449,814 (63.83% of total contacts) counselling contacts made from 2012–2018 that were greater than 60 seconds in length. Counselling contacts decreased slightly over time (APC = −0.9, 95%CI: −1.7, −0.2, *p* = 0.024; Table 1). Most counselling contacts were by phone (57.9%, *n* = 260,513) followed by webchat (25.9%, *n* = 116,558) and email (16.2%, *n* = 72,743). 

Across all media, most counselling contacts were made by teens (52.1%, *n* = 234,559) followed by young adults (35.7%, *n* = 160,714), with the fewest by children (12.0%, *n* = 54,541). As shown in Table 2, this differed by medium. Fewer teens (41.2%) made counselling contacts by phone compared to young adults (46.1%), with a greater proportion of teens making counselling contact by webchat (65.2%) compared to young adults (25.6%). Contacts by children were evenly dispersed across media.

A greater proportion of counselling contacts were made by girls (79.7%, *n* = 353,606) compared to those made by boys (18.9%, *n* = 84,044) and transgender or gender diverse people (1.4%, *n* = 6290). This breakdown of gender across counselling contacts was consistent across contact media. However, a greater proportion of email contacts were made by girls (87.1%) compared to other media, and a greater proportion of phone counselling contacts were made by boys (23.4%) compared to other media.

### 3.6. Concerns Presented during Counselling Contacts

Table 3 presents counselling contact concerns and concern groups across each medium. When counselling contacts across all media were examined, more than half were related to ‘emotional wellbeing and mental health concerns’, with suicide-related concerns and self-injury/self-harm being raised in 12% and 6% of contacts (respectively). Relationship matters were also common, with social issues (i.e., friend/peer, dating) and family relationships (i.e., child–parent, parenting, other family) being raised in 20.4% and 19.4% of contacts, respectively. As would be expected, comparisons across contact media showed that the phone was used more so to discuss most concern groups; however, when comparing the prevalence of different concern groups across media there were no notable differences (Table 3).

Table S3 presents Joinpoint trends of concerns over time, which varied by medium. For phone contacts, all concerns except emotional abuse declined from 2012–2018, consistent with the overall trend of phone counselling contacts over time. For email contacts, a decrease was observed for all concerns other than ‘offending, abusive & violent actions’. In contrast, webchat counselling contacts increased over time for all concerns, with notable increases in contacts that discussed gender/sex identification, abusive and violent actions, and practical and material assistance.

Frequencies of counselling contacts by concern groups across medium by gender and age-group are presented in Table S4. ‘Emotional wellbeing and mental health concerns’ were most common for all gender groups, followed by ‘social and family relationship-related’ problems. ‘Identity and self-concept-related’ concerns were present in more than a quarter (28.1%) of counselling contacts with youth who identified as transgender or gender diverse. This varied by contact medium, with 41.1% of email, 30.5% of webchat, and 23.9% of phone counselling contacts related to this concern area. Moreover, compared to contacts whose gender were recorded as male (48.1%) or female (56.8%), those who identified as transgender or gender diverse (63.4%) more frequently raised concerns about ‘emotional wellbeing and mental health concerns’.

Across all age groups, ‘emotional wellbeing and mental health concerns’ (>39%), and family and social relationships (>12%) were the most raised concern groups, regardless of contact medium. However, children raised ‘violence and abuse (non–family)’ in 19.2% of phone contacts, whereas this was less frequent for teens (8.9%) and young adults (4.8%).

### 3.7. Duration of Counselling Contacts

The median duration of a phone counselling contact was 32 minutes (Table S1), which was stable between 2012 and 2018 (APC = −0.17, 95%CI: −1.5,1.2, *p* = 0.757). In contrast, the total minutes of phone counselling delivered per year decreased significantly from 1,496,924 minutes in 2012 to 1,137,593 minutes in 2018 (APC= −4.19, 95%CI: −5.4, −2.9, *p* < 0.001). 

Webchat counselling contacts ranged in duration from less than one minute to 221 minutes. The median contact duration was 56 minutes, which reduced from 63 minutes in 2012 to 53 minutes in 2018 (Table S2). The decreasing median contact duration was significant between 2012 and 2015 (APC = −4.73, 95%CI: −8.0, −1.3, *p* = 0.030), but non-significant thereafter (APC = −1.24, 95%CI: −4.7, 2.3, *p* = 0.027). However, given the increased number of webchat counselling contacts, the total minutes delivered per year increased from 552,598 minutes in 2012 to 1,218,537 minutes in 2018 (APC = 12.75, 95%CI:9.5, 16.1, *p* < 0.001). In combination, the increase in the number of webchat counselling contacts and the longer average duration of a webchat in comparison to a phone contact resulted in the total time spent on counselling contacts increasing from 2,049,521 minutes in 2012 to 15,397,949 minutes in 2018, indicating a more than seven-fold increase in counselling load on KHL services.

## 4. Discussion

Young people under the age of 25 are at heightened risk of developing mental health disorders [1,2,16], and responsive mental health services are critical. This study aimed to describe how and why young people use the Australian nationwide KHL over time. Findings may be used to inform future service improvements for the KHL and related helpline services in meeting the ongoing needs of young people.

### 4.1. How are Young People Contacting the KHL Service?

The current analysis of contacts made to KHL from 2012–2018 indicates significant changes in the way young people engaged with the service. Over these years, there was an increase in the use of webchat and a decrease in use of phone and email (although the phone continued to be the most used medium for KHL contacts). Despite the total number of phone and webchat contacts (combined) decreasing per year over time, the service load provided by KHL (through counselling and information and referral) remained constant, with a total of 2,424,741 minutes in 2012 and 2,516,676 minutes in 2018. 

This is explained firstly by a reduction in the number of information and referral contacts, which tend to be brief, but minimal change in the number of counselling contacts. The reduction in the number of contacts seeking information and referral from KHL may reflect young people’s increasing access to mental health and well-being information via the internet more broadly. For instance, the KHL website was redesigned with informational content added from late 2017 onwards. Secondly, while the number of counselling contacts was stable, the time spent responding to counselling contacts increased, largely reflecting the rise in use of webchat. Indeed, while the increased use of webchat was evident for all contact types, the annual number of *counselling* webchats more than doubled from 2012 to 2018. This increase, combined with the longer duration of a webchat counselling contact (median 56 minutes) compared to a phone counselling contact (median 32 minutes), led to a greater total load on KHL services from 2012 to 2018 despite a decrease in the number of phone counselling contacts and a decrease of 10 minutes in the median counselling webchat duration. 

Increased webchat use reflects recent findings that young people prefer text-based counselling services to phone or face-to-face services [17,18]. In an Australian survey of young people (aged 15–25 years) who had contacted the KHL webchat service, the preference for text-based counselling in comparison to face-to-face services was primarily due to a perceived increase in safety and control (i.e., privacy, reduced emotional intensity, increased control and autonomy [18]). Avoidance motivations (i.e., avoiding being overheard, avoiding verbal social interactions, and minimising difficult emotions), increased perceived accessibility (i.e., speed, convenience), and perceived counselling expectations (i.e., help with low-complexity issues, realistic expectations) were also identified [18]. Additionally, a US study reported that a combined 59% of youths preferred one of three text-based services (i.e., text-messaging 25%; online chat 18.7%; social media 15.3%) compared to a phone service (41%) [19]. Emphasizing and providing ease-of-access, timeliness, and confidentiality of services from the privacy of home (or other private place) is clearly important in attracting and engaging young people in helpline services. Furthermore, the use of peer based online forums or message boards as alternative options for mental health support have also been shown to be acceptable to young people [20,21,22,23].

While there are clear reasons young people may prefer webchat services, the sector must be confident that this medium can offer similarly effective counselling. There is evidence to suggest the utility of text-based counselling. For example, after a single text-messaging counselling session from a Danish children’s helpline, 35.9% of young people reported feeling better and more than half reported having a plan of action [24]. Similarly, Navarro, Bambling et al. [18] reported young people perceived greater efficacy of text-based counselling with preferences for text-based communication (i.e., feeling heard/understood, catharsis, feeling normalised/validated/supported). Building upon these findings, Navarro and colleagues explored mental health professionals’ perspectives of factors related to higher and lower effectiveness of text-based counselling services [25]. They noted that increased complexity of presenting problems and subsequent assessment, slower response time (text vs. speaking), no non-verbal cues, and connectivity issues were factors believed to decrease service effectiveness. However, text-based communication, the counsellor’s interpersonal skills, and the use of self-management strategies were perceived as factors that would increase service effectiveness.

In response to increased use of, and some indication of preference for, text-based services, it is important that counsellors can provide appropriate support and guidance to meet young people’s needs in webchat contacts. Sindahl et al. [24] suggest appropriate text-based support includes discussing emotions, expressing empathy, and encouraging the young person to speak to someone, while Navarro, Bambling et al. [18] reported participants value feeling heard, understood, and supported during text-based contact. Moreover, given there is some indication that help-seeking by young people is low (e.g., [6]), appealing to preferred aspects of text-based counselling (e.g., safety, accessibility) [18], may increase the likelihood that young people engage with mental health services, including helplines. For instance, there may be utility in ensuring there is sufficient information and resources available online to improve awareness of helplines and the multiple avenues for contact [19]. Doing so may help increase awareness, reduce stigma, and ensure services can meet the increasing demand of young people using online services. 

It is also important to consider the financial impact of text-based services. Given the longer duration of a webchat counselling contact (as compared to telephone), increasing demand for webchat will increase costs and place financial strain on helpline services, unless efficiencies can be achieved. The 10-minute reduction in median counselling webchat duration during the study period may indicate that KHL became more efficient over time as counsellors become increasingly familiar and skilful in the provision of text-based services, but further investigation is needed to support this conclusion. 

### 4.2. Who is Contacting the KHL Service?

A vast majority of counselling contacts were made by girls, and teens or young adults rather than children or boys and gender diverse youth. These findings align with a similar study from the US showing the greatest use of a youth helpline service was by young girls aged 15–16 years, albeit that service provided peer support rather than the professional counselling provided by KHL [26]. Recent research has specifically explored barriers to help-seeking in young men and found that peers and traditional ‘masculine’ ideals were key to low mental health service utilization and tailored mental health advertising and using specific language (e.g., mental fitness) may be ways to overcome these [27], which could be applied to the helpline field. Given that many mental health disorders manifest during adolescence and young adulthood [16], and the increasing calls for greater early intervention for mental health problems, services such as KHL must be able to respond effectively to the varying severity of needs and cognitive developmental stages of children, teens, and young adults. Consequently, it is important to understand the differences in the extent of service use by different genders and age groups. Whether these differences reflect greater rates of mental health and related concerns in different demographics or differences in the likelihood of help–seeking/service utilisation requires further investigation. Nevertheless, ensuring helpline services are accessible, appealing, and relevant for any young person (regardless of age or gender) is critical. 

### 4.3. Why are Young People Contacting the KHL Service?

In terms of young people’s concerns, more than half the counselling contacts across all media related to emotional wellbeing and mental health concerns, followed by social and family relationship concerns. This is comparable with previous descriptive analyses of youth helpline services in the USA (e.g., [26]), where anxiety, stress, sadness, and depression were the most reported concerns by young people. However, while such concerns are commonly reported, we must also be mindful of the contrast in reported concerns across gender and age to adequately address those who contact the helpline. For example, in comparison to contacts from girls, contacts from boys were far less likely to relate to emotional wellbeing concerns. Contacts by youth identifying as transgender or gender diverse were more likely to discuss identity and self-concept than contacts from those identifying as male or female. Contacts about violence and abuse (non–family) were more likely to be from children than either teens or young adults. While at face value these differences may be expected, a better understanding is needed regarding why these differences exist, so that online and phone services can be targeted more effectively to specific groups of young people.

Nearly a fifth of counselling contacts included either suicide or self-injury/self-harm concerns. In numbers, this reflects 89,173 contacts between 2012 and 2018 (across all media) which equates to (on average) 34 suicide or self-injury/self-harm related contacts per day. This proportion of contacts is comparable to those reported by Kerner et al. [26] for a hotline in the USA between 2010 and 2016, with 14.2% reporting suicidal ideation and 8.2% reporting self-harm. This fits the conceptualization of helplines as a potentially useful crisis service [13]; however, future research is needed to increase understanding of the patterns of help-seeking and type of assistance preferred/required by young people at risk of suicide and/or self-harm.

### 4.4. Implications

Given the observed increase in webchat use, counsellors may need additional, specific training to maintain effective service provision through this medium, for all concern types. Further, with most counselling contacts considered complex (e.g., mental health, suicide), ongoing skill development of counsellors tailored to these areas may be useful. Similarly, tailoring the service to those using the service will be important (e.g., young women, teenagers), as well as adjusting strategies for less frequent users (e.g., young men) to ensure they are aware of, can access, and are satisfied with the services KHL provides. Interestingly, while the number of information and referral-related contacts have decreased over time, most continue to be made through the phone even though there is an enormous amount of information available online (the KHL website itself being updated toward the end of the study period). Exploring ways to continue to meet the needs of those who prefer the phone medium to source their information may be required. Lastly, the increase in webchat and total duration of counselling contacts places an increased the load on the service and this may result in financial implications for helplines in accessing and maintaining adequate funding to support this. 

### 4.5. Limitations and Future Directions

The current analysis is descriptive, which limits the conclusions that can be drawn. Nevertheless, the large nationwide database and analysis of trends over seven years of KHL usage is informative and detailed. Limitations in the data itself also affect the interpretation of findings. Importantly, analysis is of contacts not individuals and the dataset includes both first-time users of the service and young people who have contacted multiple times. This may affect obtained results around problem types and demographic information, particularly for those categories with a relatively smaller sample size. There was also substantial missing data for age and gender of clients in some analyses, and while reflective of the anonymous nature of a helpline service, needs to be considered in drawing conclusions. Interpretation of observed changes in the data may be enhanced with reference to population data to determine rates of service usage in this demographic. It is also important to note that we analyse the response data (i.e., answered contacts) as the demand for the KHL service consistently outstrips capacity to respond (response rate in 2018 was 52%) and our analysis was limited to answered contacts (response), which may have different characteristics to contact attempts (demand). Future research could also examine specific barriers and facilitators to young men accessing helplines, investigate the trajectories and needs of repeat users, and explore the effectiveness of different helpline media (webchat vs. phone). A further important consideration for future research in this field is the use of consistent terminology to differentiate services such as helplines, crisis lines, hotlines, and various online therapies such as e-therapy, web-based CBT, and mHealth, which may or may not be part of a helpline service.

## 5. Conclusions

The results revealed that from 2012–2018 there was a change in the way young people contacted KHL and the types of help being sought. There was a reduction in information seeking contacts, but overall little change in the number of contacts seeking counselling. There was a substantial increase in webchat contacts with a concomitant decrease in contacts by telephone. These findings have important implications for helpline service providers by providing indications for resource investment and training, particularly in relation to increases in online based help-seeking.

## Figures and Tables

**Table 1:** Joinpoint trend analyses across 2012–2018 for type and medium of contacts (*n* = 704,690).

	APC	LL	UL	*p*-value
Total contacts (2012–2016)	–6.5	–8.3	–14.8	0.005
Total contacts (2016–2018)	–2.2	–8.1	3.9	0.253
Counselling	–0.9	–1.7	–0.2	0.024
Information/referral	–12.6	–14.7	–10.5	0.001
Phone contacts	–9.1	–10.4	–7.8	<0.001
Counselling	–3.8	–5.4	–2.0	0.003
Information/referral	–15.6	–17.6	–13.4	<0.001
Webchat contacts	16.7	11.7	22.0	<0.001
Counselling	16.4	12.3	20.6	<0.001
Information/referral	19.1	8.4	30.9	0.005
Email contacts	–13.7	–17.1	–10.2	<0.001
Counselling	–14.0	–17.3	–10.7	<0.001
Information/referral	–12.7	–17.3	–7.8	0.001

Note: APC = annual percentage change; LL = Lower limit of 95% confidence interval; UL = Upper limit of 95% confidence interval.

**Table 2:** Contacts separated by KHL age groups, gender, and contact type (*n* = 704,690; 2012–2018).

	Phone (*n* = 467,626)		Webchat (*n* = 139,999)		Email (*n* = 97,065)		Total (*n* = 704,690)	
	*n*	%	*n*	%	*n*	%	*n*	%
**Contact Type**								
Counselling	260,513	55.7	116,558	83.3	72,743	70.0	449,814	63.8
*Percentage of all media combined*		57.9		25.9		16.2		100.0
Information and referral	207,113	44.3	23,441	16.7	24,322	30.0	254,876	36.2
*Percentage of all media combined*		81.3		9.2		9.5		100.0
**Age**								
Children	50,129	10.7	12,960	9.3	14,425	14.9	77,514	11.0
Counselling	33,270	66.4	10,655	82.2	10,616	73.6	54,541	70.4
*Percentage of media total (across all ages)*		12.8		9.1		14.6		12.1
Information and referral	16,859	33.6	2305	17.8	3809	26.4	22,973	29.6
*Percentage of media total (across all ages)*		8.1		9.8		15.7		9.0
Teens	167,998	35.9	89,371	63.8	65,416	67.4	322,785	45.8
Counselling	107,256	63.8	76,029	85.1	51,274	78.4	234,559	72.7
*Percentage of media total (across all ages)*		41.2		65.2		70.5		52.1
Information and referral	60,742	36.2	13,342	14.9	14,142	21.6	88,226	27.3
*Percentage of media total (across all ages)*		29.3		56.9		58.1		34.6
Young Adults	249,499	53.4	37,668	26.9	17,224	17.7	304,391	43.2
Counselling	119,987	48.1	29,874	79.3	10,853	63.0	160,714	52.8
*Percentage of media total (across all ages)*		46.1		25.6		14.9		35.7
Information and referral	129,512	51.9	7794	20.7	6371	37.0	143,677	47.2
*Percentage of media total (across all ages)*		62.5		33.2		26.2		56.4
	**Phone (*n* = 461,813)**		**Webchat (*n* = 138,915)**		**Email (*n* = 95,184)**		**Total (*n* = 695,912)**	
	***n***	%	***n***	**%**	***n***	**%**	***n***	**%**
**Gender (where reported)**								
Female	337,035	73.0	117,577	84.0	82,684	86.9	537,296	77.2
Counselling	193,338	57.4	98,032	83.4	62,236	75.3	353,606	65.8
*Percentage of media total (across all genders)*		75.3		84.8		87.1		79.7
Information and referral	143,697	42.6	19,545	16.6	20,448	24.7	183,690	34.2
*Percentage of media total (across all genders)*		70.1		84.1		86.1		72.9
Male	117,985	25.5	18,634	13.3	11,663	0.1	148,282	21.3
Counselling	60,158	51.0	15,354	82.4	8532	73.2	84,044	56.7
*Percentage of media total (across all genders)*		23.4		13.3		11.9		18.9
Information and referral	57,827	49.0	3280	17.6	3131	26.8	64,238	43.3
*Percentage of media total (across all genders)*		28.2		14.1		13.2		25.5
Transgender or Gender Diverse	6793	1.5	2704	1.9	837	<0.1	10,334	1.5
Counselling	3341	49.2	2275	84.1	674	80.5	6290	60.9
*Percentage of media total (across all genders)*		1.3		2.0		0.9		1.4
Information and referral	3452	50.8	429	15.9	163	19.5	4044	39.1
*Percentage of media total (across all genders)*		1.7		1.8		0.7		1.6
Gender not recorded	5813		1084		1881		8778	

Note: Percentages reflect column totals unless specified. Gender totals reflect only contact where the young person’s gender was recorded.

**Table 3:** Number and percentage of contacts across each concern type and concern group (*n* = 449,814).

Counselling Concerns	Phone		Webchat		Email		Chi^2^	*p*
	*n*	%	*n*	%	*n*	%		
Emotional wellbeing & mental health concerns	138,503	53.2	66,747	57.3	42,360	58.2	902.71	<0.001
*Emotional wellbeing*	45,253	17.4	20,501	17.6	1427	19.6	190.05	<0.001
*Loss & grief*	9514	3.7	3776	3.2	2525	3.5	40.89	<0.001
*Mental health*	61,983	23.8	30,326	26.0	16,890	23.2	269.73	<0.001
*Self-injury/self-harm*	15,685	6	10,568	9.1	7477	10.3	2043.94	<0.001
*Suicide-related*	32,264	12.4	16,552	14.2	10,560	14.5	363.06	<0.001
Family relationships	50,602	19.4	20,056	17.2	15,880	21.8	629.89	<0.001
*Child-parent relationships*	34,661	13.3	15,157	13.0	11,297	15.5	285.33	<0.001
*Changing family structures*	6993	2.7	22,121	1.8	1850	2.5	257.23	<0.001
*Other family relationships*	11,478	4.4	3962	3.4	3791	5.2	385.27	<0.001
*Parenting own children*	1060	0.4	104	0.1	61	0.1	412.70	<0.001
Social relationships	53,025	20.4	23,648	20.3	12,192	16.8	491.41	<0.001
*Friend/peer relationships*	23,044	8.8	12,977	11.1	7764	10.7	566.85	<0.001
*Dating & relationships*	31,505	12.1	11,276	9.0	4848	6.7	1909.20	<0.001
Identity & self-concept	18,447	7.1	12,078	10.4	6992	9.6	1317.54	<0.001
*Body image*	2607	1	2526	2.2	1660	2.3	1084.19	<0.001
*Cultural identity*	744	0.3	2007	0.2	138	0.2	48.18	<0.001
*Disability-related concerns*	1267	0.5	245	0.2	145	0.2	234.89	<0.001
*Gender/sex identification*	1476	0.6	10,226	0.9	502	0.7	120.09	<0.001
*Self-concept (global)*	10,761	4.1	6417	5.5	3657	5.0	375.23	<0.001
*Sexual orientation*	2310	0.9	2210	1.9	1231	1.7	767.67	<0.001
Child abuse & family violence	20,006	7.7	7009	6.0	6456	8.9	583.67	<0.001
*Exploitation by a family* *member*	43	<0.1	3	<0.1	8	<0.1	13.10	0.001
*Emotional abuse*	4515	1.7	2280	2.0	1780	2.4	157.04	<0.001
*Living in care issues*	1386	0.5	113	0.1	76	0.1	586.98	<0.001
*Neglect of child*	1123	0.4	209	0.2	216	0.3	154.47	<0.001
*Physical abuse*	9233	3.5	3560	3.1	3698	5.1	548.39	<0.001
*Sexual abuse*	4693	1.8	1403	1.2	976	1.3	215.73	<0.001
*Exposure to family* *violence*	1990	0.8	680	0.6	633	0.9	57.97	<0.001
Violence & abuse (non-family)	21,648	8.3	9245	7.9	5767	7.9	21.10	<0.001
*Bullying–school related*	11,302	4.3	5310	4.6	3601	5.0	51.03	<0.001
*Bullying–other*	2319	0.9	832	0.7	596	0.8	30.52	<0.001
*Dating & partner abuse*	1994	0.8	647	0.6	315	0.4	121.34	<0.001
*Sexual assault/abuse (non-family)*	4171	1.6	1928	1.7	877	1.2	69.23	<0.001
*Sexual harassment*	882	0.3	414	0.4	275	0.4	2.71	0.258
*Harassment & assault* *(non-sexual)*	1479	0.6	348	0.3	213	0.3	178.87	<0.001
Offending & violent actions	3082	1.2	690	0.6	421	0.6	422.02	<0.001
*Illegal/offending acts*	1741	0.7	317	0.3	150	0.2	402.92	<0.001
*Abusive actions*	1168	0.4	318	0.3	223	0.3	77.89	<0.001
*Sexual violence acts*	223	0.1	63	0.1	51	0.1	10.98	0.004
School, education & work	16,398	6.3	7929	6.8	3549	4.9	295.24	<0.001
*School authority issue*	1527	0.6	578	0.5	276	0.4	49.50	<0.001
*Employment issues*	4291	1.6	1046	0.9	448	0.6	663.57	<0.001
*Study & education*	10,867	4.2	6423	5.5	2885	4.0	391.71	<0.001
Potentially risky situations	6577	2.5	1650	1.4	934	1.3	742.82	<0.001
*Alcohol use*	1842	0.7	542	0.5	414	0.6	80.26	<0.001
*Gang/cult involvement*	88	<0.1	21	<0.1	6	<0.1	18.01	<0.001
*Drug use*	4162	1.6	891	0.8	416	0.6	765.06	<0.001
*Addictive behaviours* *(not substances)*	599	0.2	226	0.2	124	0.2	11.73	0.003
*Physical risk-taking*	165	0.1	34	<0.1	27	<0.1	21.70	<0.001
Physical & sexual health	13,918	5.3	6782	5.8	3156	4.3	197.22	<0.001
*Physical or sexual* *development*	420	0.2	290	0.2	109	0.1	38.95	<0.001
*Physical health*	7453	2.9	3157	2.7	1642	2.3	78.34	<0.001
*Sexual activity*	2874	1.1	1692	1.5	705	2.3	115.19	<0.001
*Contraception/safe sex*	397	0.2	244	0.2	78	0.1	31.42	<0.001
*Pregnancy-related* *concerns*	3074	1.2	1570	1.3	684	0.9	63.39	<0.001
Basic needs assistance	8509	3.3	1679	1.4	1036	1.4	1512.47	<0.001
*Financial assistance/concerns*	1109	0.4	301	0.3	157	0.2	108.96	<0.001
*Homelessness*	5111	2	613	0.5	447	0.6	1595.13	<0.001
*Practical/material* *assistance*	2517	1	807	0.7	457	0.6	119.37	<0.001

## Data Availability

Restrictions apply to the availability of these data. Data was obtained from yourtown (Kids Helpline) and are available to Dr Kairi Kõlves only with the permission of yourtown.

## Supplementary Materials

The following are available online at https://www.mdpi.com/article/10.3390/ijerph18116024/s1, Table S1: Descriptive statistics of the duration (in minutes) of phone counselling (*n* = 260–513) and information and referral (*n* = 207,113) contacts; Table S2: Descriptive statistics of the duration (in minutes) of webchat counselling (*n* = 118,332) and information and referral (*n* = 24,488) contacts; Table S3: Counselling concern trends across each medium in 2012–2018 (total *n* = 449,814); Table S4: Number and percentage of contacts reporting at least one problem in each problem group across gender and age groups (*n* = 449,814).
