# Supplementary material for: Help-Seeking from a National Youth Helpline in Australia: An Analysis of Kids Helpline Contacts
Source: Int J Environ Res Public Health. 2021 Jun 3;18(11):6024. doi: 10.3390/ijerph18116024 (PMC8199956; doi:10.3390/ijerph18116024)
Supplement: Supplementary file 1 [file ijerph-18-06024-s001.zip › ijerph-1237280-supplementary.pdf]

Supplementary Table S1. Descriptive statistics of the duration (in minutes) of phone counselling (n=260,513) and information and referral (n=207,113) contacts.

Year	Counselling					Information and Referral				
	Mean	Median	SD	N	Total counselling contact minutes per year	Mean	Median	SD	N	Total information and referral contact minutes per year
2012	36.69	33.38	23.14	44,253	1,496,923.52	7.27	1.92	14.65	47,693	346,592.98
2013	36.01	32.55	22.60	41,811	1,403,994.43	7.88	2.27	15.51	39,003	307,148.63
2014	35.37	32.40	21.63	42,002	1,397,377.44	6.55	2.05	13.73	32,974	216,062.14
2015	34.23	31.00	21.30	41,852	1,348,489.45	5.36	1.85	11.65	27,370	146,580.04
2016	34.81	31.72	21.64	37,092	1,220,004.06	5.55	2.28	11.01	21,765	120,890.07
2017	35.91	33.17	21.61	36,331	1,245,122.29	6.32	2.60	12.24	21,465	135,723.20
2018	35.43	32.67	21.01	33,857	1,137,593.08	6.39	2.72	11.78	16,843	107,674.49
2012-2018	35.51	32.40	21.91	260,513	9,249,514.07	6.67	2.18	13.53	207,113	1,380,580.74

*Average for mean, median and SD; total for n and counselling minutes.

**All included counselling contacts made by phone were 60 seconds or greater.

Supplementary Table S2. Descriptive statistics of the duration (in minutes) of webchat counselling (n=118,332) and information and referral (n=24,488) contacts.

Year	Counselling					Information and referral				
	Mean	Median	SD	N	Total counselling contact minutes per year	Mean	Median	SD	N	Total information and referral contact minutes per year
2012	61.25	63	23.10	9022	552,597.50	21.08	11.5	23.01	1,358	28,626.64
2013	57.79	61	21.28	12933	747,398.07	18.05	9	21.06	2,656	47,940.80
2014	53.39	57	21.42	13915	742,921.85	15.05	9	17.68	3,161	47,573.05
2015	51.74	55	22.28	16240	840,257.60	12.15	8	15.01	3,447	41,881.05
2016	50.58	54	22.86	19402	981,353.16	10.95	7	13.77	3,857	42,234.15
2017	50.81	53	23.28	20969	1,065,434.89	11.27	7	13.97	4,062	45,778.74
2018	50.61	53	21.86	24077	1,218,536.97	10.79	6	14.68	4,900	52,871.00
2012-2018	52.75	56	22.58	116558	6,148,434.50	13.09	8	16.61	23,441	306,842.69

*average for mean, median and SD; total for n and counselling minutes.

Supplementary Table S3. Counselling concern trends across each medium in 2012-2018 (total n=449,814).

	**Phone counselling concerns (n=260,513)**				**Webchat counselling concerns (n=116,558)**				**Email counselling concerns (n=72,743)**			
	**APC**	**LL**	**UL**	***p*-value**	**APC**	**LL**	**UL**	***p*-value**	**APC**	**LL**	**UL**	***p*-value**
**Emotional wellbeing & mental health**	-0.95	-2.70	0.80	.227	17.04	13.20	21.00	<.001	-13.58	-18.30	-8.60	.001
Emotional wellbeing	0.90	-2.80	4.70	.564	15.40	7.80	23.60	.003	-13.04	-18.90	-6.80	.004
Loss & grief	1.29	-19.60	27.60	.893	6.13	1.20	11.30	.024	-18.99	-25.40	-12.00	.001
Mental health concerns	-0.83	-2.80	1.20	.342	17.84	13.40	22.45	<.001	-12.48	-18.60	-5.90	.005
Self-injury/self-harm concerns	-5.83	-9.00	-2.60	.006	9.98	4.10	16.20	.007	-30.84	-57.30	11.90	.106
Suicide-related concerns	0.86	-2.90	4.80	.588	21.61	14.80	28.80	<.001	-12.80	-18.80	-6.30	.004
**Family relationships**	-5.22	-8.00	-2.30	.006	13.50	10.50	16.60	<.001	-14.10	-19.00	-8.90	<.05
**2012-2015**	*n/a*	*n/a*	*n/a*	*n/a*	*n/a*	*n/a*	*n/a*	*n/a*	-19.52	-32.90	-3.40	.036
**2015-2018**	*n/a*	*n/a*	*n/a*	*n/a*	*n/a*	*n/a*	*n/a*	*n/a*	-8.39	-23.70	9.90	.175
Child-parent relationships	-4.73	-7.80	-1.50	.013	13.62	10.20	17.20	<.001	-13.64	-17.90	-9.20	.001
Changing family structures	-5.05	-8.90	-1.10	.023	14.81	9.70	20.10	.001	-14.61	-16.90	-12.20	<.001
Other family relationships	-6.98	-10.90	2.90	.008	10.87	7.00	14.80	.001	-15.89	-19.80	-11.80	<.001
Parenting own children	-13.02	-20.70	-4.60	.012	0.08	-12.60	14.60	.988	-21.06	-34.30	-5.10	.021
**Social relationships**	1.55	-33.40	54.90	.929	10.18	5.90	14.60	.002	-18.86	-22.90	-14.60	.001
Friend/peer relationships	-6.76	-10.40	-3.00	.006	10.35	6.30	14.60	.001	-16.57	-20.40	-12.60	<.001
Dating & partner relationships	-5.46	-8.40	-2.40	.006	19.94	1.10	42.30	.041	-22.73	-28.50	-16.40	<.001
**Identity & self-concept**	-7.27	-9.30	-5.20	<.001	8.05	2.10	14.40	.017	-8.55	-44.80	51.50	.668
Body image	-10.60	-15.10	-5.80	.003	9.30	3.60	15.30	.008	-20.38	-28.50	-11.40	.003
Cultural identity	-6.76	-12.60	-0.50	.040	9.90	-4.40	26.30	.141	-13.53	-30.20	7.10	.142

Disability-related concerns	-6.13	-13.70	2.10	.112	2.18	-18.30	27.80	.814	-11.10	-22.30	1.80	.075
Gender/sex identification	6.99	-6.20	22.10	.245	47.31	28.30	69.20	.001	9.95	-0.40	21.30	.056
Self-concept (global)	-8.19	-11.30	-4.90	.001	2.18	-5.90	10.90	.530	-17.59	-27.00	-6.90	.009
Sexual orientation	-8.60	-15.30	-1.30	.030	11.42	4.10	19.30	.010	-14.75	-27.00	-4.40	.016
**Child abuse & family violence**	-4.79	-7.60	-1.80	.009	22.72	17.10	28.60	<.001	-6.50	-9.30	-3.50	<.05
**2012-2014**	*n/a*	*n/a*	*n/a*	*n/a*	*n/a*	*n/a*	*n/a*	*n/a*	-14.71	-28.20	1.30	.058
**2014-2018**	*n/a*	*n/a*	*n/a*	*n/a*	*n/a*	*n/a*	*n/a*	*n/a*	-2.06	-7.20	3.40	.242
Exploitation by a family member	-23.13	-52.10	23.40	.213	*unable to calculate due to low n*					*unable to calculate due to low n*		
Emotional abuse	5.94	1.10	11.00	.025	28.39	22.60	34.40	<.001	-0.85	-5.60	4.20	.675
Living in care issues	-13.63	-20.60	-6.00	.007	12.24	-9.10	38.50	.217	-5.50	-27.30	22.80	.200
Neglect of child	-0.42	-3.20	2.40	.716	28.56	11.90	47.70	.006	10.29	0.40	21.20	.044
Physical abuse	-2.72	-6.50	1.20	.135	24.16	15.40	33.50	.001	-5.07	-8.10	-1.90	.010
Sexual abuse	-14.39	-17.00	-11.70	<.001	13.46	5.90	21.60	.005	-11.78	-21.20	-1.20	.036
Exposure to family violence	4.77	-0.90	10.80	.085	22.15	15.60	29.10	<.001	-10.39	-18.20	-1.80	.028
**Violence & abuse (non-family)**	-3.78	-7.30	-0.10	.047	15.71	13.00	18.50	<.001	-14.12	-23.30	-3.80	.018
Bullying – school related	-3.48	-8.30	1.60	.137	16.43	13.40	19.60	<.001	-15.05	-20.50	-9.30	.001
Bullying – other	-1.95	-8.90	5.50	.522	15.79	9.80	22.10	.001	-16.43	-24.30	-7.80	.005
Dating & partner abuse	-3.30	-11.00	5.20	.400	19.94	1.10	42.30	.041	-19.59	-30.20	-7.40	.011
2012-2016	2.81	-11.30	19.20	.505	*n/a*	*n/a*	*n/a*	*n/a*	*n/a*	*n/a*	*n/a*	*n/a*
2016-2018	-14.36	-46.30	36.60	.290	*n/a*	*n/a*	*n/a*	*n/a*	*n/a*	*n/a*	*n/a*	*n/a*
Sexual assault/abuse (non-family)	-6.85	-12.80	-0.50	.039	10.56	6.10	15.20	.002	-21.10	-30.90	-9.90	.006
Sexual harassment	0.11	-2.50	2.80	.921	27.81	20.30	35.80	<.001	-10.38	-31.30	16.90	.337
Harassment & assault (non-sexual)	-4.94	-6.80	-3.10	.001	8.50	5.00	12.00	<.050	-10.89	-25.70	6.90	.165
2012-2016	*n/a*	*n/a*	*n/a*	*n/a*	15.55	9.1	22.3	.008	*n/a*	*n/a*	*n/a*	*n/a*
2016-2018	*n/a*	*n/a*	*n/a*	*n/a*	-4.43	-20.2	14.5	.393	*n/a*	*n/a*	*n/a*	*n/a*
**Offending, abusive & violent actions**	-3.81	-7.00	-0.50	.031	23.41	11.20	36.90	.004	-6.52	-14.60	2.40	.115
Illegal/offending acts	-8.34	-14.40	-1.90	.022	17.27	4.00	32.20	.019	-12.08	-28.20	7.70	.164

Abusive/violent actions	-3.55	-36.50	46.40	.833	33.84	14.90	56.00	.005	0.80	-6.10	8.20	.784
Sexual violence/offending acts	-10.84	-16.60	-4.70	.007	18.08	5.10	32.70	.015	*unable to calculate due to low n*			
**School, education & work**	-4.55	-7.40	-1.60	.011	11.38	7.10	15.90	.001	-13.41	-18.50	-7.90	.002
School authority issue	-3.72	-9.80	2.80	.198	13.83	3.30	25.40	.019	-15.70	-22.90	-7.80	.005
Employment issues	-1.14	-36.50	53.90	.949	15.43	8.00	23.40	.003	-15.26	-24.40	-5.00	.014
Study & education issues	-6.01	-10.00	-1.80	.015	10.50	6.20	15.00	.001	-12.66	-19.80	-4.80	.010
**Potentially risky situations & actions**	-8.22	-9.90	-6.50	<.001	12.50	4.30	21.40	<.050	-14.62	-20.40	-8.50	.002
**2012-2014**	*n/a*	*n/a*	*n/a*	*n/a*	27.92	-16.10	95.00	.128	*n/a*	*n/a*	*n/a*	*n/a*
**2014-2018**	*n/a*	*n/a*	*n/a*	*n/a*	5.54	-7.60	20.60	.224	*n/a*	*n/a*	*n/a*	*n/a*
Alcohol use	-5.62	-9.80	-1.30	.021	13.45	6.30	21.10	.004	-16.72	-23.00	-9.90	.002
Gang/cult involvement	-11.15	-31.00	14.50	.284	20.93	-14.80	71.60	.221	*unable to calculate due to low n*			
Drug use	-9.60	-10.90	-8.40	<.05	10.90	1.30	21.30	<.050	-13.50	-21.20	-5.00	.010
2012-2015	-6.38	-10.30	-2.30	.021	*n/a*	*n/a*	*n/a*	*n/a*	*n/a*	*n/a*	*n/a*	*n/a*
2015-2018	-12.77	-16.40	-9.00	.005	*n/a*	*n/a*	*n/a*	*n/a*	*n/a*	*n/a*	*n/a*	*n/a*
2012-2014	*n/a*	*n/a*	*n/a*	*n/a*	43.8	-13	137.6	.090	*n/a*	*n/a*	*n/a*	*n/a*
2014-2018	*n/a*	*n/a*	*n/a*	*n/a*	-2.66	-16.9	14.1	.541	*n/a*	*n/a*	*n/a*	*n/a*
Addictive behaviours (not D&A)	-3.40	-12.20	6.20	.392	15.44	7.50	24.00	.004	-4.91	-11.60	2.20	.134
Physical risk-taking	-14.15	-26.60	0.40	.054	-5.68	-34.40	35.60	.696	*unable to calculate due to low n*			
**Physical & sexual health**	-10.73	-13.10	-8.30	<.001	7.16	3.30	11.20	.005	-19.57	-26.20	-12.30	.001
Physical or sexual development	-17.65	-29.30	-4.00	.023	12.47	2.00	24.00	.027	-7.79	-19.30	5.40	.179
Physical health	-9.71	-12.40	-7.00	<.001	2.87	-2.50	8.50	.232	-20.30	-28.90	-10.70	.004
Sexual activity	-9.22	-11.60	-6.80	<.001	9.69	-0.60	21.10	.061	-15.90	-24.30	-6.60	.008
Contraception/safe sex	-13.10	-17.30	-8.80	<.05	14.30	-19.20	61.70	.400	-39.37	-49.70	-27.00	.001
2012-2015	-7.08	-20.10	8.00	.171	*n/a*	*n/a*	*n/a*	*n/a*	*n/a*	*n/a*	*n/a*	*n/a*
2015-2018	-18.79	-30.20	-5.20	.027	*n/a*	*n/a*	*n/a*	*n/a*	*n/a*	*n/a*	*n/a*	*n/a*
2012-2014	*n/a*	*n/a*	*n/a*	*n/a*	92.2	-72.10	1224.90	.283	*n/a*	*n/a*	*n/a*	*n/a*
2014-2018	*n/a*	*n/a*	*n/a*	*n/a*	-11.84	-52.10	62.30	.468	*n/a*	*n/a*	*n/a*	*n/a*

Pregnancy-related concerns	-14.16	-18.00	-10.10	<.001	12.83	4.10	22.30	.012	-22.14	-28.40	-15.40	.001
**Basic needs assistance**	-8.80	-13.30	-3.90	<.05	22.00	17.80	26.30	<.050	-14.24	-21.00	-6.90	.005
**2012-2016**	-4.01	-12.30	5.10	.191	35.74	27.70	44.30	.002	*n/a*	*n/a*	*n/a*	*n/a*
**2016-2018**	-17.54	-38.10	9.80	.102	-1.47	-18.80	19.50	.773	*n/a*	*n/a*	*n/a*	*n/a*
Financial assistance/concerns	-9.30	-17.50	-0.30	<.05	13.40	1.80	26.30	<.050	-24.22	-38.40	-6.80	.018
2012-2014	-24.45	-55.40	28.00	.149	*n/a*	*n/a*	*n/a*	*n/a*	*n/a*	*n/a*	*n/a*	*n/a*
2014-2018	-0.59	-15.80	17.40	.893	*n/a*	*n/a*	*n/a*	*n/a*	*n/a*	*n/a*	*n/a*	*n/a*
2012-2015	*n/a*	*n/a*	*n/a*	*n/a*	30.99	-6.3	83.2	.074	*n/a*	*n/a*	*n/a*	*n/a*
2015-2018	*n/a*	*n/a*	*n/a*	*n/a*	-1.89	-29.8	37.2	.830	*n/a*	*n/a*	*n/a*	*n/a*
Homelessness	-8.80	-12.80	-4.60	<.05	20.80	10.40	32.20	.003	-16.85	-23.80	-9.30	.003
2012-2016	-1.92	-9.30	6.10	.399	*n/a*	*n/a*	*n/a*	*n/a*	*n/a*	*n/a*	*n/a*	*n/a*
2016-2018	-21.10	-38.40	1.10	.054	*n/a*	*n/a*	*n/a*	*n/a*	*n/a*	*n/a*	*n/a*	*n/a*
Practical/material assistance	-8.59	-13.80	-3.10	.011	30.90	21.20	41.40	<.050	9.68	-32.60	78.40	.646
2012-2015	*n/a*	*n/a*	*n/a*	*n/a*	60.83	26.5	104.4	.014	*n/a*	*n/a*	*n/a*	*n/a*
2015-2018	*n/a*	*n/a*	*n/a*	*n/a*	6.58	-16.1	35.5	.371	*n/a*	*n/a*	*n/a*	*n/a*

*a joinpoint was identified for webchat.

** a joinpoint was identified for phone and webchat.

Supplementary Table S4. Number and percentage of contacts reporting at least one problem in each problem group across gender and age groups (n=449,814).

Counselling Problems	**Total**		**Phone**		**Webchat**		**Email**		**Chi²**	***p*-value**
	**N**	**%**	**N**	**%**	**N**	**%**	**N**	**%**		
Male										
Emotional wellbeing & mental health	40,398	48.1	28,599	47.5	7506	48.9	4,293	49.7	28.12	<.001
Family relationships	15,353	18.3	11,296	18.8	2,346	15.3	1,711	20.1	120.52	<.001
Social relationships	18,162	21.6	12,813	21.3	3,777	24.6	1,572	18.4	135.54	<.001
Identity & self-concept	7,847	9.3	5,109	8.5	1,808	11.8	930	10.9	183.14	<.001
Child abuse & family violence	5,640	6.7	4,005	6.7	831	5.4	804	9.4	141.91	<.001
Violence & abuse (non-family)	6,513	7.7	4,604	7.7	1,247	8.1	662	7.8	3.76	.153
Offending, abusive & violent actions	1,700	2.0	1,368	2.3	209	1.4	123	1.4	67.61	<.001

School, education & work	6,024	7.2	4,220	7.0	1,218	7.9	586	6.9	16.77	<.001
Potentially risky situations & actions	2,921	3.5	2,378	4.0	352	2.3	191	2.2	143.83	<.001
Physical & sexual health/development	4,264	5.1	2,985	5.0	883	5.8	396	4.6	14.49	<.001
Basic needs assistance	3,087	3.7	2,578	4.3	296	1.9	213	2.5	229.31	<.001
Female										
Emotional well & mental health	200,752	56.8	106,280	55.0	57,412	58.6	37,060	59.5	579.22	<.001
Family relationships	69,010	19.5	37,926	19.6	17,330	17.7	13,754	22.1	476.62	<.001
Social relationships	68,998	19.5	39,139	20.2	19,475	19.9	10,384	16.7	390.48	<.001
Identity & self-concept	27,416	7.8	12,332	6.4	9,424	9.6	5,660	9.1	1141.6	<.001
Child abuse & family violence	27,091	7.7	15,646	8.1	6,000	6.1	5,445	8.7	483.9	<.001
Violence & abuse (non-family)	29,257	8.3	16,462	8.5	7,810	8.0	4,985	8	32.66	<.001
Offending, abusive & violent actions	2,372	0.7	1,631	0.8	460	0.5	281	0.5	191.34	<.001
School, education & work	21,250	6.0	11,809	6.1	6,561	6.7	2,880	4.6	294.76	<.001
Potentially risky situations & actions	6,061	1.7	4,072	2.1	1,267	1.3	722	1.2	393.25	<.001
Physical & sexual health/development	19,136	5.4	10,628	5.5	5,806	5.9	2,702	4.3	191.98	<.001
Basic needs assistance	7,865	2.2	5,733	3.0	1,340	1.4	792	1.3	1078.69	<.001
Transgender or Gender Diverse										
Emotional well & mental health	3,987	63.4	2,218	66.4	1,390	61.1	379	56.2	32.96	<.001
Family relationships	826	13.1	501	15.0	236	10.4	89	13.2	25.35	<.001
Social relationships	590	9.4	294	8.8	243	10.7	53	7.9	7.68	.022
Identity & self-concept	1,769	28.1	797	23.9	695	30.5	277	41.1	92.86	<.001
Child abuse & family violence	316	5.0	101	3.0	135	5.9	80	11.9	98.18	<.001
Violence & abuse (non-family)	268	4.3	128	3.8	123	5.4	17	2.5	13.83	<.001
Offending, abusive & violent actions	65	1.0	41	1.2	16	0.7	8	1.2	3.81	.149
School, education & work	249	4.0	135	4.0	93	4.1	21	3.1	1.42	.492

Potentially risky situations & actions	55	0.9	27	0.8	24	1.1	4	0.6	1.64	.441
Physical & sexual health/development	153	2.4	91	2.7	55	2.4	7	1	6.71	.035
Basic needs assistance	63	1.0	32	1.0	30	1.3	1	0.1	7.32	.026
Children (5-12yrs)										
Emotional wellbeing & mental health	21,345	39.1	11,851	35.6	4,769	44.8	4,725	44.5	442.63	<.001
Family relationships	16,421	30.1	11,254	33.8	2,514	23.6	2,653	25	565.52	<.001
Social relationships	8,733	16.0	4,658	14.0	2,135	20.0	1,940	18.3	268.89	<.001
Identity & self-concept	2,470	4.5	937	2.8	778	7.3	755	7.1	578.98	<.001
Child abuse & family violence	6,507	11.9	4,243	12.8	999	9.4	1,265	11.9	87.62	<.001
Violence & abuse (non-family)	9,727	17.8	6,391	19.2	1,810	17.0	1,526	14.4	134.87	<.001
Offending, abusive & violent actions	308	0.6	208	0.6	44	0.4	56	0.5	6.8	.033
School, education & work	2,103	3.9	1,252	3.8	451	4.2	400	3.8	5.08	.079
Potentially risky situations & actions	494	0.9	295	0.9	84	0.8	115	1.1	5.5	.064
Physical & sexual health/development	1,574	2.9	847	2.5	419	3.9	308	2.9	55.38	<.001
Basic needs assistance	399	0.7	317	1.0	37	0.3	45	0.4	63.7	<.001
Teens (13-17yrs)										
Emotional well & mental health	126,700	54.0	53,609	50.0	42,709	56.2	30,382	59.3	1411.6 3	<.001
Family relationships	50,455	21.5	24,521	22.9	14,237	18.7	11,697	22.8	516.77	<.001
Social relationships	42,872	18.3	19,299	18.0	15,191	20.0	8,382	16.3	281.29	<.001
Identity & self-concept	20,395	8.7	6,761	6.3	8,410	11.1	5,224	10.2	1453	<.001
Child abuse & family violence	20,242	8.6	10,674	10.0	4,960	6.5	4,608	9	673.7	<.001
Violence & abuse (non-family)	19,159	8.2	9,540	8.9	5,922	7.8	3,697	7.2	152.74	<.001
Offending, abusive & violent actions	2,245	1.0	1,489	1.4	453	0.6	303	0.6	387.54	<.001
School, education & work	13,914	5.9	6,339	5.9	5,036	6.6	2,539	5	153.58	<.001
Potentially risky situations & actions	4,230	1.8	2,650	2.5	989	1.3	591	1.2	500.78	<.001

Physical & sexual health/development	13,040	5.6	6,401	6.0	4,451	5.9	2,188	4.3	209.75	<.001
Basic needs assistance	6,951	3.0	5,085	4.7	1,095	1.4	771	1.5	2171.8 9	<.001
Young adults (18-25yrs)										
Emotional well & mental health	99,565	62.0	73,043	60.9	19,269	64.5	7,253	66.8	250.83	<.001
Family relationships	19,662	12.2	14,827	12.4	3,305	11.1	1,530	14.1	74.94	<.001
Social relationships	37,260	23.2	29,068	24.2	6,322	21.2	1,870	17.2	357.73	<.001
Identity & self-concept	14,652	9.1	10,749	9.0	2,890	9.7	1,013	9.3	15.44	<.001
Child abuse & family violence	6,722	4.2	5,089	4.2	1,050	3.5	583	5.4	72.58	<.001
Violence & abuse (non-family)	7,774	4.8	5,717	4.8	1,513	5.1	544	5	8.45	.066
Offending, abusive & violent actions	1,640	1.0	1,385	1.2	193	0.6	62	0.6	84.42	<.001
School, education & work	11,859	7.4	8,807	7.3	2,442	8.2	610	5.6	77.02	<.001
Potentially risky situations & actions	4,437	2.8	3,632	3.0	577	1.9	228	2.1	125.82	.001
Physical & sexual health/development	9,242	5.8	6,670	5.6	1,912	6.4	660	6.1	33.58	<.001
Basic needs assistance	3,874	2.4	3,107	2.6	547	1.8	220	2	65.76	<.001
